# All You Need Is Evidence: What We Know About Pneumonia in Children With Neuromuscular Diseases

**DOI:** 10.3389/fped.2021.625751

**Published:** 2021-09-01

**Authors:** Claudio Cherchi, Maria B. Chiarini Testa, Daniele Deriu, Alessandra Schiavino, Francesca Petreschi, Nicola Ullmann, Maria G. Paglietti, Renato Cutrera

**Affiliations:** ^1^Pediatric Pulmonology and Respiratory Intermediate Care Unit, Sleep and Long Term Ventilation Unit, Academic Department of Pediatrics (DPUO), Pediatric Hospital “Bambino Gesù” Research Institute, Rome, Italy; ^2^Rare Diseases and Medical Genetics Unit, Bambino Gesù Children Hospital (IRCCS), Rome, Italy; ^3^Department of Pediatrics, University of Rome Tor Vergata, Rome, Italy

**Keywords:** neuromuscular disease, SMA, duchene muscular dystrophy, pneumonia, atelectasia

## Abstract

Neuromuscular diseases may involve all major respiratory muscles groups including inspiratory, expiratory, and bulbar muscles. Respiratory complications are the major cause of morbidity and mortality. Pneumonia represents a frequent cause of morbidity in children with neuromuscular disease. The aim of this review is to collect knowledge about pneumonia in children with neuromuscular diseases. Pneumonia usually follows viral respiratory infections of the upper respiratory tract, due to the combination of an increased amount of nasal and oral secretions and an impairment of the cough efficiency and of the clearance of secretions due to the muscle weakness, further compromised by the infection itself. The accumulation of bronchial secretions leads to atelectasis and promote bacterial infection. Moreover, dysfunction of swallowing mechanism exposes these children to the risk of developing aspiration pneumonia. However, etiology of viral and bacterial respiratory infection in these patients is still poorly studied.

## Introduction

Neuromuscular diseases (NMD) may involve most respiratory muscles groups including inspiratory, expiratory, and bulbar. Respiratory complications are the major cause of morbidity and mortality. The natural course of NMD is characterized by ineffective cough and swallowing disorders leading to chronic aspiration, poor secretion clearance, pneumonia and hypercapnic respiratory failure.

Pneumonia represents a frequent cause of morbidity in children with NMD; inhalation, impaired cough and atelectasis are the main risk factors. However, scarce literature investigating on pneumonia in children with NMD is available.

This review is part of the research topic “Emerging Pneumonia in Children” and its aim is to summarize knowledge about pneumonia in children with neuromuscular diseases and to encourage research in this neglected field.

## Respiratory Involvement in Neuromuscular Disease

NMD are a heterogeneous group of diseases characterized by lesions that may involve motor,neurons, peripheral nerve, neuromuscular junction, or skeletal muscle. They consist of acquired and inherited forms, characterized by very variable age of onset, clinical features, courses and prognoses (1). Most are inherited forms as Duchenne muscular dystrophy (DMD), spinal muscular atrophy (SMA), congenital muscular dystrophies and myopathies. NMD clinical features range from profound floppiness and respiratory compromising at birth, such as patients affected by SMA type 1, to mild motor impairment and late-onset respiratory problems.

The incidence of respiratory complications varies according to diagnosis, genotype and age (for details, see the British Thoracic Society guideline for respiratory management of children with neuromuscular weakness) (2).

NMD are characterized by a restrictive pattern of spirometry on pulmonary function testing (3). This is caused by the presence of reduced inspiratory muscle strength, thoracic scoliosis and reduced chest wall and pulmonary compliance. Usually, inspiratory and expiratory muscle strength are equally impaired, however in SMA the diaphragm strength may be preserved and expiratory muscle weakness may prevail (4). Progressive neuromuscular weakness can lead to the inability to take deep breaths and to cough effectively (5).

Cough efficacy can be measured by cough peak flow: reduced cough power is defined by values <270 L/min in children, and values <160 L/min are associated with high risk of atelectasis and pneumonia (6).

Respiratory muscle weakness is frequently unrecognized in children and NMD remain undiagnosed until ventilatory is precipitated by aspiration pneumonia or acute respiratory airway infection (7). Acute respiratory failure due to accumulation of lung secretions often represents the first clinical relevant manifestation of these diseases (5). Although the progression of symptoms may be predictable, the timeline will vary, according to the type of NMD and the age of the patient: an infant with SMA type I would be expected to experience serious respiratory impairment, leading to infectious complications within the first year of life, a patient affect by DMD probably would not experience pneumonia before his second decade of life (5). NMDs characterized by recurrent pneumonia are shown in Table 1.

**Table 1:** Neuromuscular diseases with recurrent pneumonia.

Spinal muscular atrophy type 1
Duchenne muscular dystrophy
SMA with respiratory distress type 1
Facioscapulohumeral muscular dystrophy with infantile onset
Myotonic dystrophy 1 with severe congenital onset
Charcot marie tooth with severe early onset
Pompe disease

## Pathophysiology of Pneumonia in Neuromuscular Diseases

Pneumonia in children with NMD usually follows viral respiratory infections, even if confined to the upper airway (8). Acute upper respiratory infections can cause atelectasis or pneumonia by several mechanisms. It is well-known that viral upper respiratory infections can cause an acute worsening in respiratory muscle strength in the healthy adult population (7). In children affected by NMD the further reduction of an already compromised respiratory muscle strength can cause shortness of breath, decreased vital capacity and acute hypercapnia (2). Moreover, the infections are often associated to an increased production of nasal and oral secretions. Nasopharyngeal secretions also become thicker and purulent, thus weakening an already compromised swallowing mechanism and leading to an higher risk of aspiration of infected upper airway secretions into the lower respiratory tract. The impaired cough mechanism, deriving from the respiratory muscle weakness, makes those secretions more difficult to clear from the lower airways. Children with NMD are particularly prone to develop segmental or lobar atelectasis due to the retention of bronchial secretions. This is particularly evident in the lower lung zones, which may already be compressed by scoliosis or by the heart. They are also at risk for developing widespread, radiographically inapparent micro-atelectasis (8). The appearance of atelectasis exposes these patients to a higher risk of bacterial lung superinfections (9).

Children presenting swallowing disorders, ineffective cough, atelectasis and capacity vital forced (FVC) <50% of predicted value are at high risk of pneumonia and should be trained in protocols that allow successful home treatment of respiratory exacerbations, performed by well-trained parents or healthcare professionals (10, 11).

**Figure 1:**
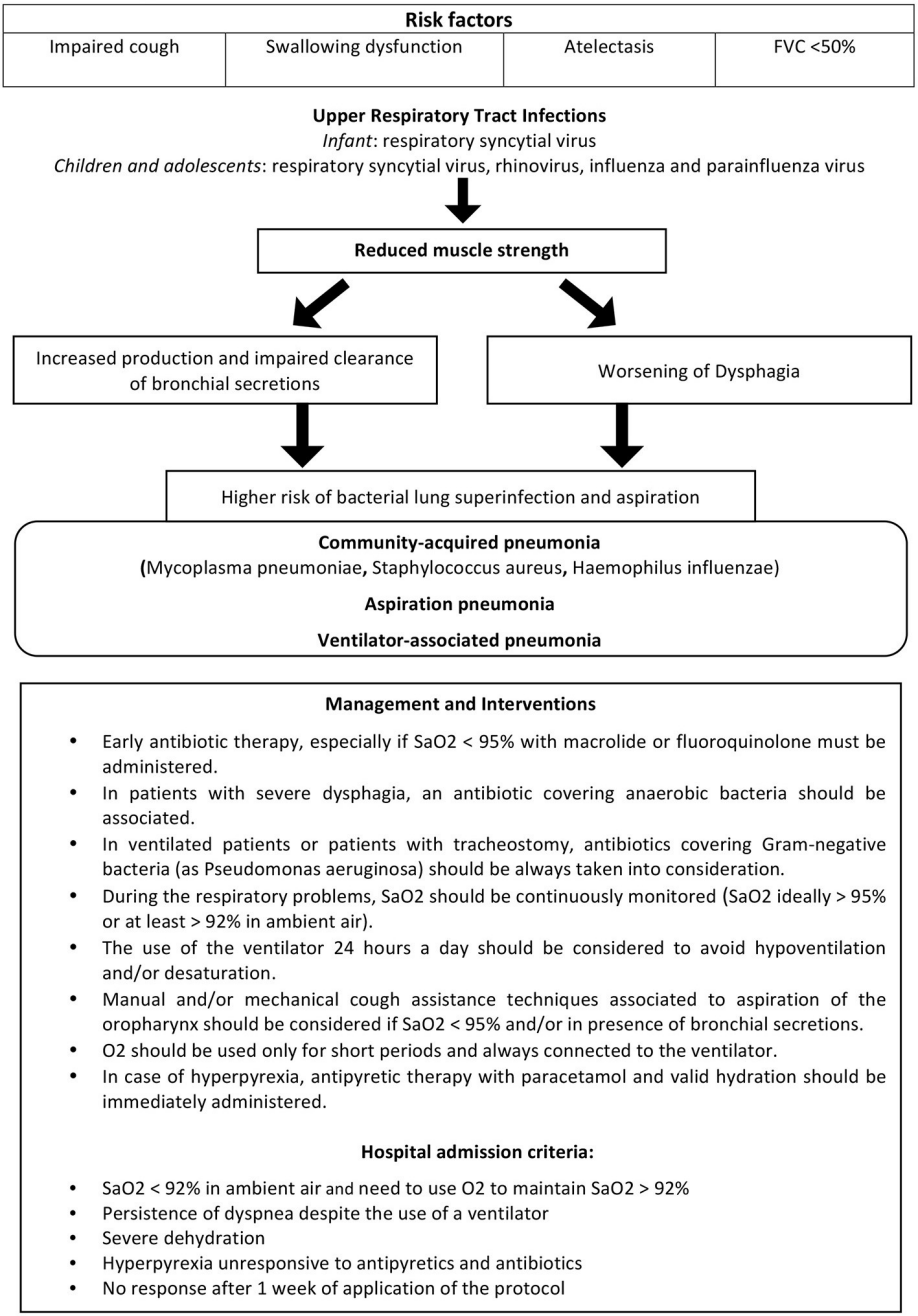
Pathophysiology, clinical management and treatment of respiratory exacerbations/pneumonia in NMD.

Risk factors, pathophysiology and treatment of respiratory exacerbations and pneumonia in patients with NMD are shown in Figure 1.

### Community-Acquired Pneumonia

As specified before, pneumonia in children with NMD usually follows viral respiratory infections (12). Generally, respiratory syncytial virus (RSV) infection predominates in infants, while rhinovirus, influenza and parainfluenza virus infections are common in children and adolescents (2). The risk of serious pulmonary complications associated to RSV infection led the American Academy of Pediatrics to introduce NMDs in the list of diseases requiring RSV immuno-prophylaxis (9). However, scarce literature investigating on microbiologic etiology of both viral and bacterial pneumonia in children with NMD is available (12).

Few data come from a cohort study conducted on 28 children with NMD admitted to PICU (13) which found both viruses (respiratory syncytial virus, rhinovirus, influenza virus) and bacteria (Mycoplasma pneumoniae, Staphylococcus aureus, Haemophilus influenzae) as etiological agents associated to acute respiratory infections, defined by a positive culture from airways within 48 h of admission or signs of upper respiratory tract, respiratory symptoms, fever, and/or leukocytosis (12).

### Aspiration Pneumonia

Children with NMD show swallowing dysfunction that increases as muscle weakness progresses, and in some conditions such as SMA type 1 and severe forms of nemaline myopathy may present from early infancy (2). Difficulties in swallowing, resulting from loss of control of the larynx and pharynx, associated to ineffective cough predispose to aspiration lung disease (2). Aspirated material includes saliva, mouth organisms and food in children orally fed (2). Gastric contents may be aspirated if gastro-esophageal reflux is present. Aspiration causes inflammation of the lung parenchima and obstruction of the lower airways, leading to worsening restrictive lung disease, Kooi-van Es. et al. (14) demonstrated that the overall prevalence of dysphagia, in a large group of children with NMD was 47.2% (13). Although oral and pharyngeal weakness can increase the risk of aspiration lung disease, aspiration is a relatively rare event and an uncommon cause of respiratory exacerbation (2).

However, for children who cannot safely achieve an adequate oral nutrition, the positioning of a percutaneous gastrostomy should be considered to improve quality of life and reduce respiratory complications.

### Ventilator-Associated Pneumonia

Ventilator-associated pneumonia (VAP) is a common hospital-acquired infection and a source of increased morbidity (15). Between 3 and 19% of all ventilated children are diagnosed with VAP at a reported frequency of 1.1–27.1 per 1,000 ventilator days (16). Due to the high incidence of PICU admissions among children affected by NMD, VAP represent a frequent infective complication and a serious threat. However, literature about this setting of patients is completely lacking.

## Clinical Management and Treatment of NMD Pediatric Patient With Respiratory Exacerbations and Pneumonia

Development of respiratory exacerbations may be a life-threatening event in patients with NMD, deriving from secretion accumulation and further weakening of respiratory muscles, and leading to acute respiratory failure (17). In clinical practice, the management includes early or prophylactic use of antibiotics for respiratory exacerbations, although no studies proving the benefits of this approach are available in literature (2). An antibiotic therapy should be started early, especially when SaO_2_ is <95% and the antibiotic coverage must include atypical bacteria (macrolide or fluoroquinolone) (17). In case of suspected inhalation (e.g., in patients presenting with severe dysphagia), a second antibiotic for anaerobic bacteria should be associated (e.g., amoxicillin associated with clavulanic acid) (17). Antibiotics covering Gram-negative bacteria (as Pseudomonas aeruginosa) should be always taken into consideration for ventilated patients or patients with tracheostomy (18). Home management of a respiratory tract infection should comprise a daily or at least every 2–3 days visit of the patient, performed by the specialist or the general practitioner, to prescribe antibiotic therapy and exclude the presence of hospital admission criteria suggested by Racca et al. (17) (shown in Figure 1). The general practitioner should maintain telephone contact with a specialist with expertise in home ventilation in order to share the decision-making process (17).

During the infectious exacerbation, the value of SaO_2_ should be continuously monitored by pulse oximetry and SaO_2_ should be ideally maintained >95% or at least >92% in ambient air.

The use of the ventilator 24 h a day should be considered to avoid hypoventilation and/or SaO_2_ < 95%.

When the value of SaO_2_ falls below 95%, especially in presence of bronchial secretions the use manual and/or mechanical cough assistance techniques must be considered.

In younger children and in patients with severe dysphagia, the use of a cough machine should always be followed by secretion aspiration in the oropharynx with the aid of a mechanical aspirator.

Oxygen (O_2_) therapy can be provided for short periods and the oxygen source must be always connected to the ventilator to prevent hypoxia and hypercapnia. Finally, fever >38.5°C must be treated with paracetamol and a valid hydration protocol should be followed (17).

Finally, during respiratory exacerbations children with NMD are often exposed to chest X-ray which could be reduced if a noninvasive and reliable diagnostic method is identified (17). The use of lung ultrasound should be recommended for early identification of pulmonary atelectasis, in order to reduce frequent ionizing exposition of these fragile patients (19).

## Discussion and Future Perspectives

Pulmonary complications represent a frequent cause of morbidity and mortality in patients affected by NMD. The pathophysiology of respiratory exacerbations in patients with NMD is deeply understood. Pneumonia usually follows viral respiratory infections of the upper respiratory tract, due to the combination of an increased amount of nasal and oral secretions and an impairment of the cough efficiency and of the clearance of secretions due to the muscle weakness, further compromised by the infection itself. The accumulation of bronchial secretions leads to atelectasis and promote bacterial infection. Moreover, dysfunction of swallowing mechanism exposes these children to the risk of developing aspiration pneumonia.

However, etiology of viral and bacterial respiratory infection in these patients is still poorly studied. No significant data, investigating the etiological agents involved in respiratory infections of children with NMD are available in literature.

A precise characterization of microbial etiology of pneumonia in NMD pediatric patients would be of great help in the antimicrobial strategy definition. Evidence-based empiric antibiotic therapy guidelines for these patients would ameliorate the clinical management of respiratory exacerbations. Wide studies, aimed at identifying the most frequent pathogens involved in community-acquired pneumonia and correlation between etiology and mortality, are necessary to help physicians in their clinical practice. Colonization of the lower respiratory tract, through deep cough-produced sputum culture should also be investigated on large cohorts of children with NMD.

Recently, the possibility of an early diagnosis of NMD, through the introduction of neonatal screening programs, associated to the availability of innovative therapeutic approaches, consisting of Nusinersen for SMA and gene therapy for SMA and DMD, are completely changing the natural history of these diseases (20). Life expectancy of these children drastically improved, exposing them to a higher number of complications. Literature focused on respiratory assistance need to keep up with times.

## Author Contributions

CC contributed to the research and critical evaluation of the available literature and wrote the first draft of the manuscript. MC, DD, AS, and FP contributed to the research and critical evaluation of the available literature and to writing sections of the manuscript. NU and MP contributed to the research and critical evaluation of the available literature and manuscript. RC contributed to writing the first draft of the manuscript. All authors read and approved the final manuscript.

## Conflict of Interest

The authors declare that the research was conducted in the absence of any commercial or financial relationships that could be construed as a potential conflict of interest.

## Publisher's Note

All claims expressed in this article are solely those of the authors and do not necessarily represent those of their affiliated organizations, or those of the publisher, the editors and the reviewers. Any product that may be evaluated in this article, or claim that may be made by its manufacturer, is not guaranteed or endorsed by the publisher.
